# How keratin cortex thickness affects iridescent feather colours

**DOI:** 10.1098/rsos.220786

**Published:** 2023-01-11

**Authors:** Deok-Jin Jeon, Seungmuk Ji, Eunok Lee, Jihun Kang, Jiyeong Kim, Liliana D'Alba, Marie Manceau, Matthew D. Shawkey, Jong-Souk Yeo

**Affiliations:** ^1^ School of Integrated Technology, Yonsei Institute of Convergence Technology, Yonsei University, Incheon 21983, Republic of Korea; ^2^ Department of Research Planning, National Institute of Ecology, Chungcheongnam-do 33657, Republic of Korea; ^3^ Ecological Technology Research Team, Division of Ecological Applications Research, National Institute of Ecology, Chungcheongnam-do 33657, Republic of Korea; ^4^ Evolution and Optics of Nanostructures Group, Department of Biology, Ghent University, Ledeganckstraat 35, Ghent 9000, Belgium; ^5^ Naturalis Biodiversity Center, Darwinweg 2, Leiden 2333 CR, The Netherlands; ^6^ Center for Interdisciplinary Research in Biology, CNRS UMR7241, INSERM U1050, Collège de France, Paris Sciences et Lettres University, 75006 Paris, France

**Keywords:** structural colour, iridescence, keratin cortex, avian coloration

## Abstract

The bright, saturated iridescent colours of feathers are commonly produced by single and multi-layers of nanostructured melanin granules (melanosomes), air and keratin matrices, surrounded by an outer keratin cortex of varying thicknesses. The role of the keratin cortex in colour production remains unclear, despite its potential to act as a thin film or absorbing layer. We use electron microscopy, optical simulations and oxygen plasma-mediated experimental cortex removal to show that differences in keratin cortex thickness play a significant role in producing colours. The results indicate that keratin cortex thickness determines the position of the major reflectance peak (hue) from nanostructured melanosomes of common pheasant (*Phasianus colchicus*) feathers. Specifically, the common pheasant has appropriate keratin cortex thickness to produce blue and green structural colours. This finding identifies a general principle of structural colour production and sheds light on the processes that shaped the evolution of brilliant iridescent colours in the common pheasant.

## 1. Introduction

Colour-producing mechanisms in nature can broadly be divided into two categories: pigmentary colours and structural colours [1–4]. Most blue, violet, ultraviolet and some green colours in nature are structural colours [5–7], which are the result of light being reflected by nanostructures that have a feature size comparable to visible wavelengths (i.e. in hundreds of nanometers). Unlike pigmentary colours, optical nanostructure can produce colours via wavelength-selective interference of light. Constructive interference, diffraction and scattering of light are the main physical phenomena that produce structural colours [8,9]. Structural colours are common in nature, found in organisms ranging from birds [5,7,10–19] and insects [20–23] to land plants [24,25] and even algae [26].

In feathers, iridescence is a unique optical property of structural colour [27,28]. A feather typically comprises a central rachis with serial paired branches (i.e. barbs) possessing further branches (i.e. barbules). Iridescent colours can be produced by multi-layers of nanostructured melanin granules (melanosomes) within a keratin matrix in feather barbules. Thus, iridescent colours depend strongly on the morphology of the nanostructures in feather barbules, which determines how the refractive index varies. More saturated colours can be produced by increasing the difference between the refractive indices, the degree of order or the thickness of optical nanostructures [29–32]. Moreover, the varying shapes of the melanosomes (solid rods, hollow rods, solid platelets or hollow platelets) also play a significant role in producing structural colours, as they can vary refractive index profiles [32–35].

Although the effects of melanosomes in producing iridescent colours are relatively well understood, the effects of a keratin cortex in combination with melanosomes are unclear. Previous studies reported that keratin cortex can play a major role in producing iridescence under the weak influence of either poorly ordered melanosomes [36] or single-layered melanosomes [37]. In the case of multi-layered melanosomes, Stavenga *et al*. [38] have shown that the hue (i.e. the position of the major reflectance peak) of feathers is most sensitive to the changes in a multi-layer period and cortex thickness using an optical multi-layer model. Their theoretical results also suggest that cortex thickness can be responsible for the overall saturation and brightness. These studies shed light on the significance of keratin cortex on structural plumage coloration. However, it remains to be seen whether birds actually adjust their plumage colour by evolving different cortex thicknesses. Here, we investigate the iridescent blue and green feathers of the common or ring-necked pheasant (*Phasianus colchicus*) by performing plasma-etching on the cortex to provide empirical confirmation of the theoretical predictions to answer this question.

## 2. Materials and methods

### 2.1. Optical measurements

Common pheasant feathers were obtained from road-kills at the Chungnam Wild Animal Rescue Center and the Ulsan Metropolitan City Wildlife Rescue and Management Center in Korea. We cut blue and green feathers on the neck and then washed them in a solution of 0.1% Tween 20 and ethanol [11].

**Figure 1.**
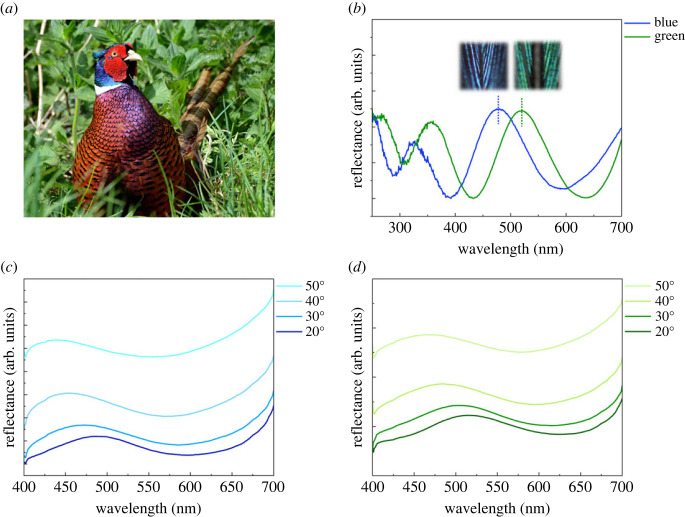
Optical properties of the feathers of a common pheasant. (*a*) A male common pheasant (*Phasianus colchicus*) in its natural habitat. This bird has blue and green feathers on its neck. Photo credit: Pam Parsons. (*b*) Diffuse reflectance spectra of blue and green feathers. The dotted lines indicate reflectance peaks at 480 nm for blue and 520 nm for green feathers. Specular reflectance curves for incident angles from 20° to 50° for (*c*) blue and (*d*) green feathers.

The diffuse and specular reflectance of bird feathers was measured using an ultraviolet–visible (UV-Vis) spectrophotometer (Cary 5000, Agilent Technologies) for UV-Vis wavelengths (250–700 nm) (figure 1). To measure the diffuse reflectance, we used the diffuse reflectance accessory, which contains an integrating sphere. To measure the specular reflectance, we used the variable-angle specular reflectance accessory, which rotates the sample. In the specular reflectance measurements, the light was polarized with the polarizing filter (Filter Polariod Vertical/Horizontal HN 32, Agilent Technologies). The specular reflectance spectra were obtained by averaging results from vertically and horizontally polarized light. Since the polarizing filter does not have authorized data in the UV region, we analysed the reflectance spectrum for incident angles ranging from 20° to 50° in the visible range.

We used a microspectrophotometer (20/20 PV, Craic Technologies) to measure the reflectance of microscale barbules. To fix the location of the barbules during etching and the reflectance measurement, we attached the feathers to a carbon tape. The spectra were collected for visible wavelengths (350–750 nm) by focusing on an area of 16.3 × 16.3 µm with a 52 × objective lens under normal incidence illumination using a Xe lamp.

### 2.2. Structural characterization

The nanostructure of the feathers was characterized by transmission electron microscopy (TEM). We prepared the TEM samples as follows. The feathers were fixed by dipping them in a mixture of 2% (v/v) glutaraldehyde and 2% (v/v) paraformaldehyde in 0.05 M cacodylate buffer (pH 7.2) for 4 h. The feathers were washed with the 0.05 M cacodylate buffer solution. Post-fixation was performed with 1% OsO_4_ in the 0.05 M cacodylate buffer for 1 h. Then, the feathers were dehydrated by agitating them consecutively in 30% to 100% ethanol solutions. The feathers were embedded in resin (LR white resin, Sigma-Aldrich) at 50°C for 24 h. Ultrathin sections (80–100 nm thick) were prepared using an ultramicrotome with a diamond knife. The thin sections were stained with uranyl acetate and lead citrate and then examined using a TEM (JEM-2100F, JEOL).

**Figure 2.**
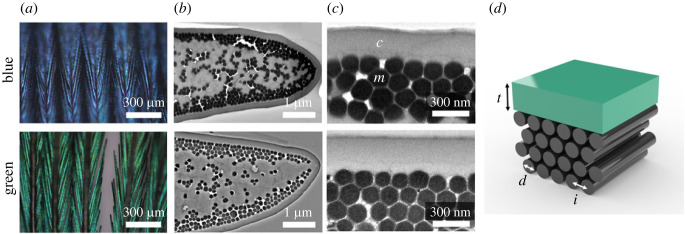
Structure of common pheasant feathers. (*a*) Optical micrographs of blue and green barbules. (*b*) TEM images of nanostructures in blue and green barbules. (*c*) Corresponding magnified TEM images, showing the keratin cortex (*c*) and melanosomes (*m*). The keratin cortex above the multi-layered and highly ordered melanosomes. (*d*) Schematic illustration, showing structural parameters (cortex thickness (*t*), melanosome diameter (*d*) and interspatial distance (*i*)).

From the TEM images, we classified the structures with three parameters: (i) the thickness of the keratin cortex, (ii) the diameter of a melanosome, and (iii) the interspatial distance (figure 2). The thickness of the keratin cortex was defined as the distance between the outermost edge of the barbule and the outermost melanosome layer. The interspatial distance was defined as the distance between the centre of a melanosomes and the centre of another melanosome (i.e. pitch).

We obtained average values of these parameters from at least 16 images using ImageJ software. We measured at least 480 points for the average radius of melanosomes, 101 points for the average interspatial distances and 64 areas for the average thickness of keratin cortices in blue and green barbules. All measurements were conducted in areas without any distortions by an ultramicrotome. To characterize the diameter of melanosomes, circular-shaped melanosomes (only parallel cut of rod-shaped melanosomes can show circular shape in two-dimensional TEM images) were selected and then analysed. Cortex thickness was measured at the vertex and the covertex of ellipsoidal barbules.

To verify the significance, *f*-test was applied to evaluate the equality of two-sample variances as a preliminary test which enabled to determine the type (i.e. equal or unequal variance) of *t*-test. Then, we used two-tailed *t*-tests to compare variables (cortex thickness, diameter of melanosome and interspatial distance) between blue and green groups using Microsoft Excel.

### 2.3. Etching of the keratin cortex

We etched the keratin cortex in the feather barbules using oxygen plasma, which refers to a plasma treatment performed while oxygen gas is introduced into the plasma chamber (ASS-5000, SNTek). This method is good for etching organic materials, and it does not leave any residue. We etched feather barbules with a fixed power (50 W), working pressure (400 mTorr) and oxygen gas flow (40 standard cubic centimetres per minute (SCCM)). Starting from original (oxygen plasma untreated) feather barbules, we controlled the thickness of the keratin cortex by varying the etching time.

To measure variations of the keratin cortex thickness after the etching process, we obtained cross-sectional images of the etched barbules with scanning electron microscopy via focused ion beam (FIB) milling. Before FIB milling, tungsten and platinum were deposited as a passivation layer to minimize the damage caused by the ion beam to the edge of the barbules.

### 2.4. Optical simulations

To gain an in-depth understanding of the effects of the thickness of the keratin cortex, we performed optical simulations and compared these results with empirical reflectance data. We used the finite-difference time-domain (FDTD) method using a commercial software (Ansys Lumerical FDTD). We generated separate honeycomb geometries consisting of rod-shaped melanosomes for the blue and green feathers. The simulation models were based on the empirical observations in table 1. The multi-layered melanosomes are surrounded by the keratin matrix. By changing the thickness of the keratin cortex, the interspacing distance and the diameter of melanosomes, reflectance spectra are obtained through the detector. The distances between the source and the detector, the source and the nanostructure are 200 and 2000 nm, respectively. To acquire all the light reflected by the structures, we applied periodic boundary conditions along the *y*-axis, which was parallel to the layers of melanosomes and perfectly matched layers to the ends of *y* points (*y*_min_ and *y*_max_) (electronic supplementary material, figure S1). From the simulation, we obtained reflectance spectra for visible wavelengths (400–700 nm) for keratin cortices of different thicknesses.

**Table 1.** Means (s.d.) of structural parameters for barbules from blue and green feathers (two-sample *t*-tests, all *p* < 0.05) where *N* indicates the sample size.

structural parameter	blue	green
thickness of the keratin cortex (nm)	259.53 (30.30)_, *N*__=__64_	283.59 (30.05)_, *N*__=__80_
diameter of a melanosome (nm)	138.54 (15.58)_, *N*__=__480_	125.04 (14.13)_, *N*__=__600_
interspatial distance (nm)	150.06 (13.60)_, *N*__=__101_	156.49 (15.46)_, *N*__=__117_

Previous studies assumed that the refractive index of melanin is 2.0; however, more recent empirical evidence suggested that the refractive index varied from 1.7 to 1.8 depending on the wavelength [39–41]. Thus, we applied that the wavelength-dependent refractive index of melanin to optical simulations and the refractive index of keratin is 1.55 [42,43]. A complex refractive index of melanin was applied in the model to account for absorption. Compared with melanosomes, the absorption by the keratin matrix can be considered negligible.

## 3. Results

### 3.1. Optical measurements

Optical measurements indirectly indicate the structural origin of the colours produced by the feathers of the common pheasant. The common pheasant has brilliant blue and green colours on its neck (figure 1*a*). In the diffuse reflectance spectra (figure 1*b*), these blue and green feathers have prominent peaks at 480 and 520 nm, respectively. The specular reflectance spectra of the blue (figure 1*c*) and green (figure 1*d*) feathers depend on the viewing angle. Whereas pigmentary colours are independent of angle, angle-dependent colours (i.e. iridescent colours) are generally considered to be structural. For the blue and green feathers, as the incident angle (i.e. tilt angle) increased from 20° to 50°, the total reflectance increased and there were large blue shifts (i.e. hypsochromic shifts) of both reflectance peaks (figure 1*c,d*). Thus, the angle-dependent optical properties strongly indicate that neck feather colours are structural and not pigmentary.

### 3.2. Structural characterization

Structural characterization showed differences in the structural parameters between the barbules from blue and green feathers. The optical micrographs show that the colours come from the barbules and not the barbs (figure 2*a*). Therefore, we focused on characterizing the structures in the barbules using TEM. As expected, the TEM images for both the blue and green barbules showed that the crystalline multi-layers comprised solid melanosomes, which we call ‘multi-layered melanosomes' (figure 2*b*,*c*). Both sides of a barbule had the multi-layered melanosomes and the keratin cortex. Through image processing of the TEM images, we calculated the structural parameters (the thickness of the keratin cortex, the radius of a melanosome and the interspatial distance) of the blue and green barbules (figure 2*d* and table 1).

The green barbules had thicker keratin cortices than the blue barbules, whereas the blue barbules had larger melanosomes than the green ones. There were only slight differences in the interspatial distances (approx. 6 nm) of blue and green barbules. The radius of a melanosome and the interspatial distance affect the structural colour because they change the refractive index profile of the crystalline melanosome layers [34]. By contrast, the thickness of the keratin cortex affects the structural colour because it provides a refractive index gradient in combination with the melanosomes and causes thin film interference [15,16,36,39,44]. Specifically in our study, green barbules had cortices on average 24 nm thicker relative to those of blue barbules, which is approximately half the difference between the reflectance peaks for blue and green feathers (40 nm). Consequently, this indicates that the keratin cortex could play a major role in the colours produced by common pheasant feathers, as it affects the constructive interference of the reflected light.

### 3.3. Etching of the keratin cortex

**Figure 3.**
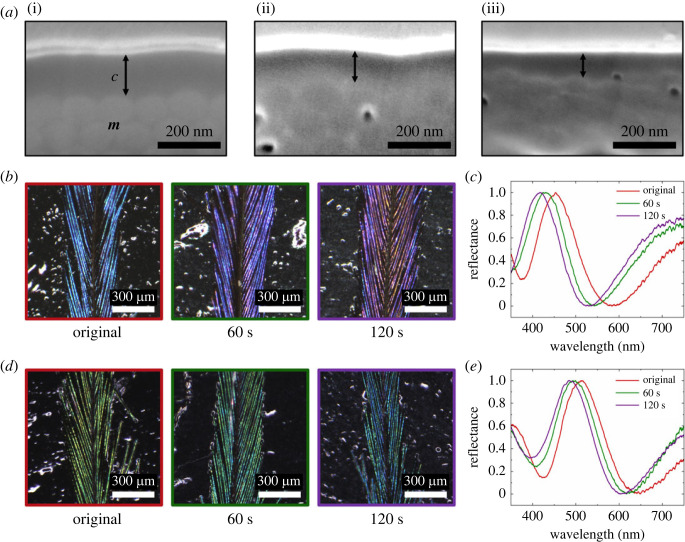
Colour changes after etching the keratin cortex. (*a*) Keratin cortices with different thicknesses after etching of a blue barbule. Electron micrographs of the barbule etched with an oxygen plasma for 0 s (original) (i), 60 s (ii) and 120 s (iii), respectively. The solid arrows represent the keratin cortex thicknesses (keratin cortex (*c*) and melanosome (*m*)). (*b*) Optical micrographs of original blue barbules and barbules etched for 60 or 120 s. (*c*) Corresponding normalized reflectance spectra of blue barbules. (*d*) Optical micrographs of original green barbules and barbules etched for 60 or 120 s. (*e*) Corresponding normalized reflectance spectra of green barbules. Compared with original barbules (red boxes and lines), the reflectance peaks of barbules etched for 60 s (green boxes and lines) or 120 s (purple boxes and lines) had blue shifts. Unnormalized reflectance spectra are shown in the electronic supplementary material.

To test more directly the effect of cortex thickness on the resulting coloration, we used an oxygen plasma treatment to etch the keratin cortex. By setting the length of the oxygen plasma treatment, we controlled the decrease in the thickness of the keratin cortex (figure 3*a*). While these FIB-SEM images in figure 3*a* have clearly shown variations of keratin cortex thicknesses after etching barbules, the thickness of keratin cortex is slightly distorted by the passivation layer as well as the imaging of curved barbule at a given tilt angle.

By changing the thickness of the keratin cortex, the colours of the barbules were changed (figure 3*b–e*). Compared with the original blue barbules, the reflectance spectra of barbules etched for 120 s had shifted to shorter wavelengths, becoming violet (figure 3*b*). There were clear blue shifts in the peaks of the reflectance spectra, going from 455 to 430 and to 418 nm on changing the etching time (figure 3*c*; electronic supplementary material, figure S2A). As for the blue barbules, the green barbules also dramatically changed in colour, going from green to blue-green on increasing the etching time and decreasing cortex thickness (figure 3*d*). The peaks of the normalized reflectance spectra also clearly had a blue shift, going from 514 to 497 and to 485 nm (figure 3*e*; electronic supplementary material, figure S2B). These results demonstrate that plumage colours can change with direct modifications of the thickness of the keratin cortex.

Because of the distortion of the cortex thickness in FIB-SEM images, the change in cortex thickness could not be directly compared with the results from TEM observations of pheasant barbules. Additionally, the curved barbules could not be uniformly etched since the plasma-based method etched the surface in a direction perpendicular to the substrate. Thus, the results are qualitative rather than quantitative due to these experimental constraints. Our optical simulations will provide more quantitative understanding to the experimental findings in the following section.

### 3.4. Optical simulations

To evaluate the quantitative effects of the thickness of the keratin cortex, we used optical simulations. As we observed experimentally, the thickness of the keratin cortex regulated the colours of feather barbules. Our simulations were thus conducted based on empirical geometries for both types of nanostructures found in blue and green barbules.

**Figure 4.**
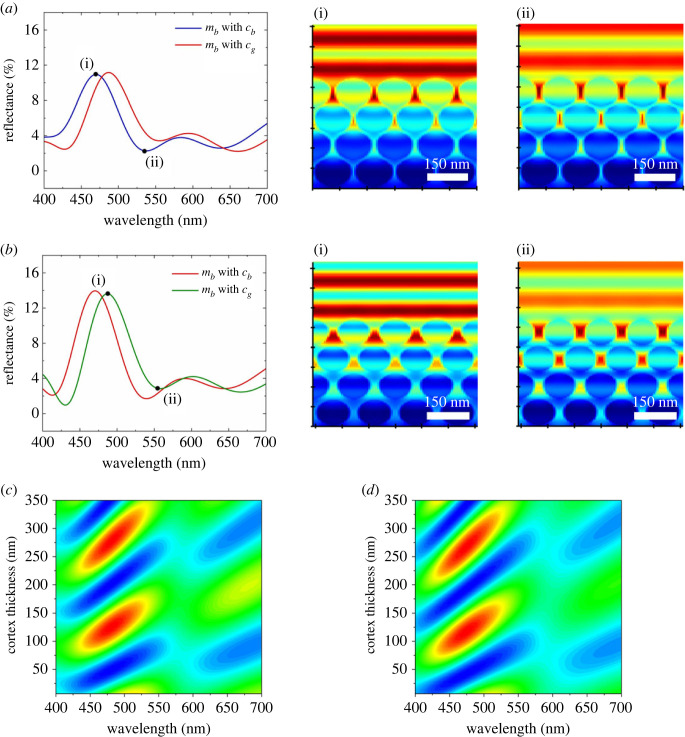
Results of optical simulations of the effects of the thickness of the keratin cortex. (*a*) Reflectance spectra of blue melanosomes (*m*_b_) with a blue keratin cortex (*c*_b_) or a green keratin cortex (*c*_g_). E-field distributions showing (i) high reflectance at 470.3 nm and (ii) low reflectance at 535.7 nm. Black dots indicate the points where e-field distributions were obtained for *m*_b_ with *c*_b_. (*b*) Reflectance spectra of green melanosomes (*m*_g_) with a green keratin cortex (*c*_g_) or a blue keratin cortex (*c*_b_). E-field distributions showing (i) high reflectance at 489.6 nm and (ii) low reflectance at 556.2 nm. Black dots indicate the points where e-field distributions were obtained for *m*_g_ with *c*_g_. Colour contour plots of the reflectance spectra against the thickness of the keratin cortex for (*c*) blue and (*d*) green structures. Blue: minimum, red: maximum.

First, we compared the reflectance spectra of multi-layered melanosomes from blue barbules (*m*_b_) surrounded by a keratin cortex (*c*_b_) with the reflectance spectra of the same melanosomes but surrounded by the thicker keratin cortex from green barbules (*c*_g_) (figure 4*a*). This change in the thickness of the keratin cortex resulted in a clear shift in the reflectance peak from 470.3 to 489.1 nm. Then, we compared the reflectance spectra of multi-layered melanosomes from green barbules (*m*_g_) surrounded by a keratin cortex from green barbules (*c*_g_) with the reflectance spectra of the same melanosomes but surrounded by the thinner keratin cortex from blue barbules (*c*_b_) (figure 4*b*). Again, there was a clear shift in the reflectance peak from 489.6 to 471.1 nm. Moreover, the electric field (e-field) distributions for the four configurations show that only triple layers of melanosomes are highly involved in the reflectance. Stavenga *et al*. [38] have shown that the impact on hue diminished as the number of multi-layer stacks increased. Our finding concurs with the previous study. These optical modelling results suggest that keratin cortex thickness can play a major role in determining the hue even when feathers have multi-layered melanosomes.

We varied the thickness of the keratin cortex from 0 to 350 nm for both melanosomes in the blue barbule (figure 4*c*) and green barbule (figure 4*d*). A comparison of the two panels shows that there are two regions with a red shift (i.e. a bathochromic shift): (i) thicknesses from 100 to 150 nm and (ii) thicknesses from 225 to 325 nm. For the common pheasant, there was a red shift (480 to 520 nm) of the reflectance peak upon increase of the thickness of the keratin cortex (260 to 284 nm). These simulation results confirm empirical results, clearly revealing the relationship between the thickness of the keratin cortex and the reflectance peak.

## 4. Discussion

We investigated the under-explored role of keratin cortex surrounding multi-layered melanosomes in the common pheasant feather barbules. We found that changing the cortex thickness could play a major role in changing colour, even without making other structural changes. Specifically, our empirical and simulation results have convincingly shown that the thickness of the keratin cortex could determine the hue, in concert with the underlying reflector array.

Most previous research has studied the effects of changing the thickness of the keratin cortex on the glossiness of black barbules [44], iridescence from poorly ordered melanosomes [36] and iridescence from single-layered melanosomes [37]. Some studies have suggested that the keratin cortex surrounding multi-layered melanosomes affects the colours of bird feathers [16,37,38,45]. Of these, most have considered keratin cortices of different thicknesses with theoretical models [36–38,46], statistical analyses [33,45] and by observing barbule samples where the keratin cortex had been accidentally removed [36,37]. Specifically, based on a quantitative optical modelling of multi-layered melanosomes in mallard barbules—a very similar system to the pheasant feathers examined in this study—Stavenga *et al*. [38] have convincingly explained the effect of changing barbule cortex thickness on the iridescent colours produced by multi-layered melanosomes on the hue. In the relatively recent work, Freyer *et al*. [37] have suggested cortex thickness is a key for the plumage colour from single-layered melanosomes by applying effective-medium multi-layer modelling, and the melanosomes play only a minor role. Our direct experimental evidence on altering the thickness of the keratin cortex around multi-layered melanosomes clearly indicates that the keratin cortex could govern the feather colour.

From our experimental results, we could hypothesize that the thickness of the keratin cortex affects the hue. However, a question still remained whether changing cortex thickness affects other optical properties such as saturation and brightness. In the previous study, Stavenga *et al*. [38] have shown that an untuned cortex can cause a sub-peak reducing an overall saturation and brightness. To quantitatively validate this hypothesis, optical simulations were conducted. We analysed the change in simulated reflectance spectrum by changing keratin cortex thickness for the blue and green melanosomes (figure 4). Our simulation results suggest that the change in the cortex thickness alone can lead to the change in the position of the reflectance peak, and therefore, the keratin cortex is a key in determining the hue of the pheasant feathers. Furthermore, we found that the cortex is changing within the range where a sub-peak does not occur considerably as shown in figure 4*d*,*e*, making it robust to changes in the overall saturation and brightness.

To clarify the effect of keratin cortex on determining the hue, we also simulated reflectance spectra for only the keratin cortex layer (electronic supplementary material, figure S3). Unlike feather barbules (figure 1*b*) and our simulations with a cortex and melanosomes (figure 4), the colours produced by the cortex alone appeared less saturated (electronic supplementary material, figure S3) and had different reflectance peak positions. Indeed, the multi-layered melanosomes are not completely independent of determining the hue. In our study species, the coloration system consists of a thin keratin cortex surrounding multi-layered melanosomes, where an array of closely packed and highly ordered melanosomes resembles a honeycomb photonic crystal. In this system, the multiple layers of melanosomes could act as a Bragg reflector. When the periodicity of melanosomes and the keratin cortex are under 300 nm thick (they are comparable to the wavelength of visible wavelengths), the keratin cortex could possibly result in a structure with a refractive index gradient [39], leading to constructive or destructive interference [45,47]. Our results suggest that the presence or absence of multi-layered melanosomes has a significant impact on the hue; however, changes of melanosomes in pheasant barbules are not sufficient to affect the hue.

Theoretically, the refractive index of the melanosomes could also affect the iridescent colours of bird feathers. In fact, the refractive index of melanosomes has been a controversial parameter in previous studies. Whereas many studies assumed that the refractive index of a melanosome is 2.0 [39,40,48], some studies reported that the refractive index varied from 1.7 to 1.8 depending on the wavelength [41,49]. Those studies suggested that the overly high refractive index of melanosomes could have a strong influence on the optical properties of multi-layered biophotonic structures [16,37]. In this study, the optical simulations were conducted using most recently reported empirical data for the refractive index of the melanosomes.

In our simulations, we assumed that the array of multi-layered melanosomes was perfectly ordered to systematically analyse the effects of the keratin cortex. In fact, the array had size variations and disorder in structures. To assess the effects of this assumption on the coloration mechanisms of the common pheasant feathers, we ran optical simulations using a structure imported from a TEM image (electronic supplementary material, figure S4). As shown in figure 4, the reflectance peaks simulated for the blue and the green feathers are 20 nm less than the results from the optical measurement of the common pheasant feathers. The difference in the shape and the arrangement between actual melanosomes and perfectly ordered melanosomes in the simulation could have contributed to the reflectance peak difference. However, this difference does not alter our conclusion that the thickness of the keratin cortex has a role in determining the hue of bird feathers.

We have verified iridescent colours of the common pheasant feathers result from a combination of multi-layered melanosomes and keratin cortex based on quantitative optical modelling and direct experimental observation. Whereas multi-layered melanosomes played a major role in determining saturation and maximum reflectance, keratin cortex played a major role in determining reflectance peak position.

Interestingly, we found that the common pheasant has appropriate thickness of the keratin cortex to produce blue and green structural colours, in concert with the underlying melanosomes. According to the optical modelling, the thickness of the keratin cortex should be approximately in the 100–150 nm or 225–325 nm range (figure 4*c*,*d*) to produce bright visible colours. We observed that the common pheasant has 260–284 nm thick keratin cortices (table 1), and the thickness of the keratin cortices in the range of 100–150 nm may not be observed possibly due to mechanical reasons considering the size of common pheasant. Based on the results, we have shown that the pheasant can adjust the hue by evolving different cortex thicknesses. Our finding provide valuable insights that even slight nanoscale differences in the thickness of the keratin cortex can cause significant changes to the reflectance peaks.

These results suggest that evolutionary processes have led to an ingenious structural way to produce brilliant iridescent colours in the common pheasant. Understanding the efficient design solutions found by nature for structural coloration mechanisms is desirable, as these solutions may suggest optimal designs for various nanophotonic applications.

## Data Availability

All data needed to evaluate the conclusion in this paper are available in the main text or the electronic supplementary material [50].
